# The Prevalence and Determinants of Long-Term Glycemic Control Based on Serial Glycosylated Haemoglobin Values in Type 2 Diabetes Mellitus

**DOI:** 10.7759/cureus.83221

**Published:** 2025-04-29

**Authors:** Maya Tholiya, Mahadev Meena, Rajnish Joshi, Shubham Atal, Ankur Joshi, Mohit Awasthi, Yasmee Khan

**Affiliations:** 1 General Medicine, All India Institute of Medical Sciences (AIIMS), Bhopal, IND; 2 Internal Medicine, All India Institute of Medical Sciences (AIIMS), Bhopal, IND; 3 Pharmacology, All India Institute of Medical Sciences (AIIMS), Bhopal, IND; 4 Community and Family Medicine, All India Institute of Medical Sciences (AIIMS), Bhopal, IND; 5 Internal Medicine, All India Institute of Medical Sciences (AIIMS), BHOPAL, IND

**Keywords:** determinants, glycaemic control, glycaemic variability, hba1c, type 2 diabetic mellitus (t2dm)

## Abstract

Introduction: Diabetes mellitus has become a significant public health issue in India, with a considerable proportion of the population living with the condition. Type 2 Diabetes Mellitus (T2DM), commonly linked with obesity, is associated with metabolic abnormalities and vascular complications. Effective long-term glycemic control is important in preventing complications and improving patient outcomes.

Objective: To examine associations between demographic, anthropometric, clinical, and therapeutic characteristics and long-term glycemic control status in individuals with T2DM in 314 participants.

Methodology and results: This is a longitudinal study conducted to evaluate factors such as demographic characteristics, clinical parameters, duration of T2DM, medication use, and comorbidities. Findings highlight the impact of age, gender, obesity, medication adherence, and comorbid conditions on glycemic control.

Conclusion: A multi-faceted approach, including lifestyle modifications, personalized treatment plans, and patient education, is essential for achieving optimal glycemic outcomes in T2DM management. ​

## Introduction

Per the 2019 International Diabetes Federation (IDF), 77 million people were living with diabetes in India in 2019 [1]. The age-adjusted prevalence of type 2 diabetes mellitus (T2DM) is 10.4% in the age group of 20 to 79 years [1-2]. Most of the diabetic patients have obesity-related T2DM, which is strongly associated with metabolic abnormalities and angiopathic complications [3]. Poor glycemic and blood pressure control have an additive adverse impact on the vascular endothelium, which significantly increases the risk of microvascular and macrovascular complications such as retinopathy, nephropathy, neuropathy, coronary heart disease, and stroke [4]. Recent estimates from India suggest that about 80% of individuals with T2DM have hemoglobin A1c (HbA1c) levels above 7%, indicative of glycemic control [5].

Glycemic control is affected by various factors, including age, body mass index (BMI), duration of diabetes, family history, high blood pressure, and comorbidities such as hypertension or dyslipidaemia [6,7]. Additionally, optimization of pharmacological and non-pharmacological therapies by care providers and adherence to these interventions also play an important role in glycemic control [7]. Both sustained and optimal glycemic control are desirable outcomes. Long-term glycemic status is measured using HbA1c. Although an HbA1c value less than 7.5% is desirable in most patients, targets may differ and depend on age and the presence of diabetes-specific complications [7].

Beyond glycemic control, glycemic variability (GV) has been associated with cardiovascular outcomes. Recent evidence suggests that GV affects various organs through oxidative stress, glycation, chronic low-grade inflammation, endothelial dysfunction, platelet activation, impaired angiogenesis, and renal fibrosis [8]. Additionally, oscillating hyperglycemia contributes to beta cell dysfunction. Long-term glycemic management has two dimensions: control and variability. Our research aimed to study whether adult individuals with T2DM who are consistently well-controlled (compared to the poorly controlled) have a different distribution of demographic, anthropometric, disease, and therapy-related variables.

## Materials and methods

Patients with diabetes mellitus have been followed up at the medicine outpatient department (OPD) at All India Institute of Medical Sciences (AIIMS), a tertiary care hospital in Bhopal, India, since its inception in 2013. Outpatient care of these patients is organized by the Department of Medicine through a specialty clinic. Over the years, about 1164 patients have been registered in this clinic. Since 2018, a digital record of visits and outpatient prescriptions has been maintained. During the COVID-19 pandemic, physical clinics were disrupted, and patient follow-ups were conducted via telemedicine. Since August 2021, once a week, this clinic has provided face-to-face consultations and online video-conferencing-based consultations, respectively. A record of diagnostic tests, comorbidities, prescriptions, and adherence information is maintained by this clinic. The HbA1c of the OPD patients is measured every three months.

This is a retrospective cohort study of T2DM patients between 2018 and 2023 with a prospective follow-up. We identified eligible participants from the existing records, and informed consent was obtained from each patient. We conducted a questionnaire-based interview for eligible participants, following written informed consent. All study procedures were initiated after obtaining permission from the Institutional Human Ethics Committee, Post Graduate Research, All India Institute of Medical Sciences (AIIMS), Bhopal (approval no. IHEC-PGR/2022/PG/Jan/20).

Inclusion and exclusion criteria

**Figure 1:**
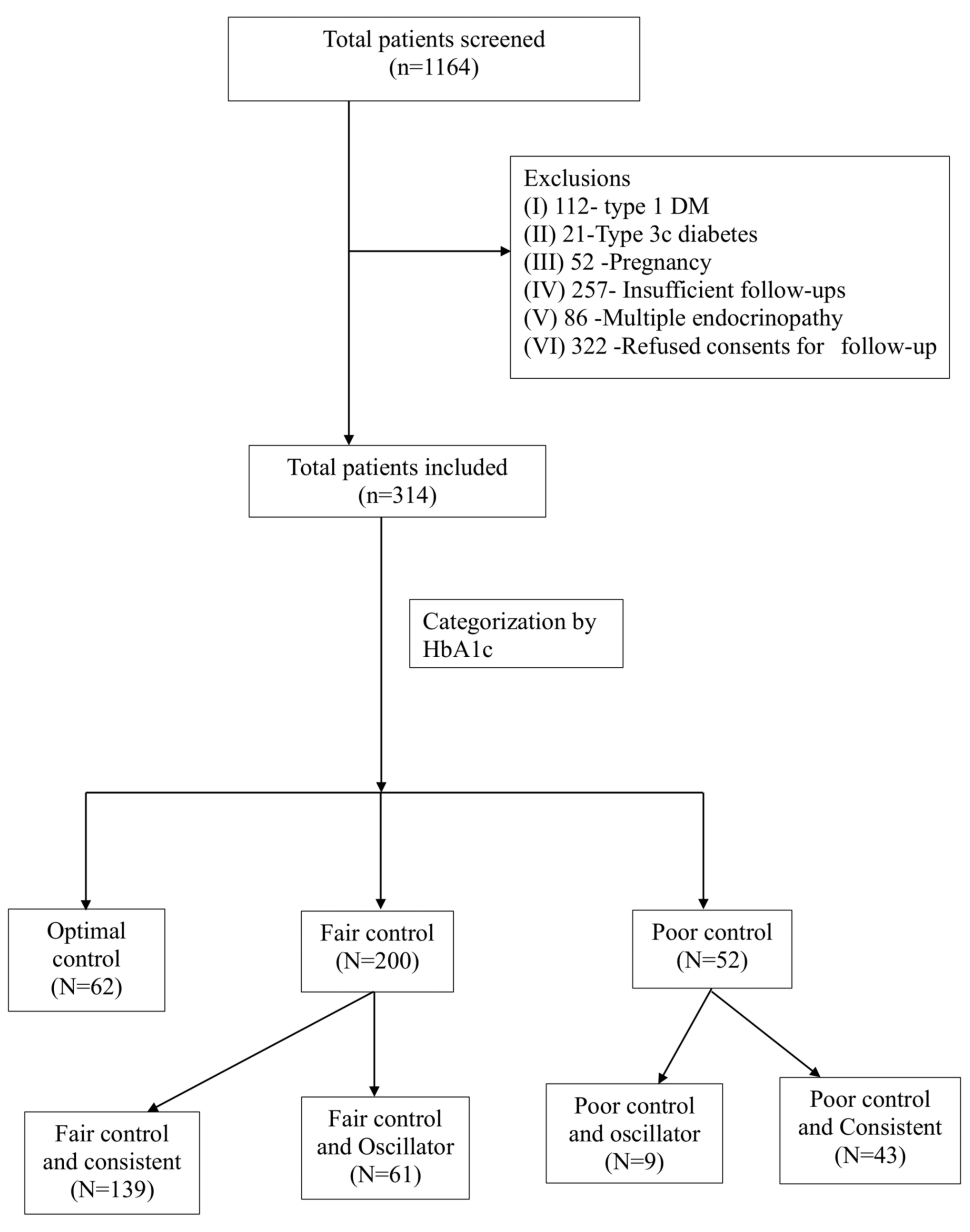
Flowchart on screening of participants

All individuals registered for the diabetes follow-up clinic were screened for inclusion. We included adults aged 35 years and older who were diagnosed with T2DM, who were registered at the diabetes follow-up clinic, and had at least three outpatient or telemedicine visits since January 2018. We excluded participants who had type 1 DM; pregnancy at any time during the study period; insufficient follow-up, such as fewer HbA1c values for interpretation; multiple endocrine conditions; type 3c diabetes; and those who did not provide consent for follow-up. Of a total of 1164 patients who were registered for follow-up at the diabetes clinic, we included 314 individuals in the present study (Figure 1).

Procedures

We collected data from the diabetes clinic using a structured questionnaire. Patients were interviewed for demographic details, addiction history, including tobacco chewing, and smoking. Clinical measurements, including height, weight, abdominal obesity, and blood pressure, were recorded. Patient records were reviewed to obtain relevant medical histories like duration of diabetes, documented complications, comorbidities, and prescribed therapies (oral hypoglycemic drugs, anti-hypertensive drugs, lipid-lowering drugs, anti-platelet drugs). Laboratory results, including plasma glucose values (fasting or random), HbA1c, creatinine, and albuminuria from urine analysis, were extracted from patient records. During follow up we collected similar information from case records.

Statistical analysis

All data was collected using data-abstraction forms and entered into MS Excel (Microsoft Corp., Redmond, WA, USA). The entered data was cleaned, and key variables were checked for consistency before analysis. Glycemic control subgroups were key effect modifiers, and GV was a key outcome. These variables were secondarily determined based on glycemic control variables obtained at different time points. The glycemic control classification was determined based on serial HbA1c values for each individual. Optimal control was defined as the average and range of all HbA1c values below 7%. Fair control was defined as an average HbA1c value between 7.1% and 9%. Poor control was defined as HbA1c values above 9%. We further sub-classified the fair-controlled category as “consistent” if the range of values was also between 7.1% and 9%. If the range included values that were less than 7.1% or more than 9%, these were “oscillators”. Similarly, we subclassified the poor control category as “consistent” if the range of HbA1c values was always above 9% and “oscillators” if the range also included lower values. We compared the distribution of the collected parameters across the above glycemic control categories. All dichotomous variables were compared using the chi-square test, all continuous variables were compared using the student t-test, and median were compared using the Wilcoxon rank sum test. A p-value of less than 0.05 was considered statistically significant. All statistical analysis was done using the statistical software R (R Foundation for Statistical Computing, Vienna, AUT).

## Results

The participants were followed up from 2018 onwards, and the last follow-up was done in 2023. During this follow-up, information about their HbA1c levels was collected. The mean HbA1c value of all participants was 7.95 ± 1.13 % (range 5.70 to 12.55%). Based on the HbA1c value, we classified patients into five groups (Table 1). Only 62 (19.74%) patients had optimally controlled diabetes, 139 (44.26%) had diabetes fairly controlled and consistent, 61(19.42%) patients were fairly controlled but were highly variable, and 52 (16.55%) patients had poorly controlled diabetes.

**Table 1:** Classification of patients based on HbA1c and mean HbA1c values HbA1c: Hemoglobin A1c

Parameter	Definition	No. of patients N (%)	Mean HbA1c (SD)
Optimally controlled	The average of all HbA1c values is below 7%, and the range of values is also below 7.	N=62 (19.74%)	6.64 (0.309)
Fairly controlled - Consistent	The average of HbA1c values is between 7.1 and 9.0%, and the range of values is between 7.1 and 9.	N=139 (44.26%)	7.80 (0.522)
Fairly controlled - Oscillators	The average of HbA1c values is between 7.1 and 9.0%, and the range of values is beyond 7.1 and 9.	N=61 (19.42%)	8.01 (0.062)
Poorly controlled- Oscillators	The average of HbA1c values is above 9.0%, and the range of values includes less than 9.	N=9 (2.86%)	9.37 (0.058)
Poorly controlled - Consistent	The average of HbA1c values is above 9.0%, and the range of values is also consistently above 9.0	N=43 (13.69%)	10.00 (0.88)

Most participants were middle-aged (average age 56.89 years, range 24 to 83 years). Of the 314 participants, 137 (43.6%) were female and 177 (56%) were male. Twenty-two (7 %) patients were smokers. The average duration of diabetes was 10 years or longer. At baseline, the most common complication was neuropathy, followed by nephropathy, and overall, microvascular complications were more than macrovascular complications. Hypertension was found in 91 (28%) individuals. The majority of the patients, i.e., 222 (71%), were taking metformin, and 159 (50.7%) patients were on sulfonylurea (Table 2).

**Table 2:** Baseline characteristics (recorded at the time of first enrolment at the diabetes follow-up clinic) of the participants (total n=314) SBP: Systolic blood pressure, DBP: Diastolic blood pressure, DPP4: Dipeptidyl peptidase 4, SGLT2: Sodium-glucose cotransporter-2, ACEI: Angiotensin-converting enzyme inhibitor, ARB: Angiotensin receptor blockers

Parameter	Value N (%)
Mean age (SD) in years	56.89 (12.55)
Female gender, n (%)	137 (43.6%)
The average duration of DM (SD) in years	10.85 (8.26)
Tobacco chewing, n (%)	39 (12.4%)
Smoking, n (%)	22 (7.0%)
Retinopathy, n (%)	7 (2.22%)
Neuropathy, n (%)	23 (7.32%)
Nephropathy, n (%)	10 (3.18%)
Known coronary artery disease, n (%)	9 (2.8%)
Known previous stroke, n (%)	6 (1.91%)
Hypertension, n (%)	91 (28.9%)
Thyroid dysfunction, n (%)	22 (7.00%)
Average BMI (SD) in kg/m2	26.01 (4.05)
Average SBP (SD) (mmHg)	137.01 (17.77)
Average DBP (SD) (mmHg)	77.04 (10.35)
Average serum creatinine (SD)	0.79 (0.23)
Metformin, n (%)	222 (70.9%)
Sulphonylurea, n (%)	159 (50.7%)
DPP4 inhibitors, n (%)	77 (24.6%)
Pioglitazone, n (%)	35 (11.2%)
SGLT2 inhibitors, n (%)	12 (3.8%)
Insulin, n (%)	32 (10.22%)
Statins, n (%)	80 (25.4%)
Low dose aspirin, n (%)	80 (25.4%)
ACEI/ARB, n (%)	99 (31.5%)

Our findings (Table 3) indicate that glycemic control is generally better in older age groups compared to younger ones. The group with poor glycemic control has a higher proportion of females and a higher average BMI. Individuals with a longer duration of diabetes tend to have poorer glycemic control. The prevalence of complications, such as retinopathy, neuropathy, nephropathy, coronary artery disease (CAD), and previous strokes, is notably higher in those with poorer glycemic control. Notable differences in the usage of insulin, DPP4 inhibitors, and pioglitazone are seen in participants with optimally controlled versus those with poor control and consistent control. Data suggests that there are differences in demographic and clinical characteristics across the different levels of control.

**Table 3:** Characteristics of participants by glycemic control and variability classification G: Group, CAD: Coronary artery disease, DPP4: Dipeptidyl peptidase 4, SGLT2: Sodium-glucose cotransporter-2, ACEI: Angiotensin-converting enzyme inhibitor, ARB: Angiotensin receptor blockers A statistically significant difference in DPP4 inhibitors(p=0.04), pioglitazone (p=0.05), and insulin (p=0.001) use was seen in participants who were optimally controlled versus those with poor control and consistency. *p-value < 0.05

Parameter	Optimally controlled and consistent( Group-I)	Fairly controlled and consistent (Group-II)	Fairly controlled and oscillating (Group III	Poorly controlled and oscillating (Group-IV)	Poorly controlled and consistent (Group-V)	p-value G-I/G-V)
Number	62 N(%)	139 N(%)	61 N(%)	9 N(%)	43 N(%)	
Mean age (SD) in years	59.70 (12.08)	57.80 (10.81)	54.75 (11.87)	53.55 (14.58)	53.72 (12.43)	0.74
Female gender n (%)	23 (37.09%)	58 (41.72%)	25 (40.98%)	5 (55.5%)	26 (60.46%)	0.006'*'
Average BMI (SD) in kg/m2	25.65 (3.62)	26.03 (4.18)	25.79 (4.35)	28.26 (4.73)	26.37(3.65)	0.95
Average SBP (SD) (mm Hg)	138.53 (14.97)	139.49 (17.84)	133.82 (17.69)	131 (16.23)	133 (21.27)	0.03'*'
Average DBP (SD) (mm Hg)	76.5 (11.08)	78.05 (10.11)	76.58 (9.58)	89.57 (12.76)	74.27 (10.54)	0.87
Average duration of DM (SD) in years	10.64 (8.41)	10.40 (6.98)	11.33 (10.63)	9.77 (6.26)	12.13 (8.52)	0.71
Retinopathy	1 (1.61%)	1 (0.71%)	3 (4.91%)	0	2 (4.65%)	0.364
Neuropathy	6 (9.67%)	10 (7.19%)	3 (4.91%)	1 (11.11%)	3 (6.97%)	0.455
Nephropathy	3 (3.22%)	2(1.43%)	4 (6.55%)	0	2 (4.65%)	0.543
Known CAD	0	4 (2.87%)	2 (3.27%)	0	3 (6.97%)	0.06
Known previous stroke	1 (1.61%)	1 (0.71%)	3 (4.91%)	0	1 (2.32%)	0.654
Hypertension	15 (24.19%)	42 (30.21%)	17 (28.86%)	2 (22.22%)	15 (34.88%)	0.165
Metformin n (%)	44 (70.96%)	95 (68.34%)	48 (80.3%)	7 (77.77%)	27(64.28%)	0.335
Sulphonylurea	28 (45.16%)	69 (69.34%)	34 (55.73%)	6 (66.67%)	22 (52.38%)	0.261
DPP4 inhibitors	13 (21.31%)	31 (24.46%)	14 (22.95%)	2 (22.22%)	17 (39.53%)	0.04'*'
Pioglitazone	5 (8.06%)	9 (6.56%)	12 (19.97%)	1 (11.11%)	8 (19.04%)	0.05'*'
SGLT2 inhibitors	2 (3.22%)	6 (4.31%)	3 (0.49%)	0	1 (2.32%)	0.636
Insulin	2 (3.22%)	13 (9.35%)	4 (0.65%)	0	13 (30.23%)	0.001'*'
Statins	19 (30.64%)	32 (23.02%)	16 (26.2%)	1 (11.11%)	12 (27.90%)	0.468
Low-dose aspirin	15 (24.19%)	25 (17.98%)	18 (29.5%)	1 (11.11%)	12 (27.90%)	0.320
ACEI/ARB	18 (29.03%)	46 (33.09%)	21 (34.2%)	2 (22.22%)	12 (27.90%)	0.540

Overall, 17 (24.41%) participants who were optimally controlled had a low complication rate, and 22 (42.31%) who were poorly controlled (oscillating and consistent) had a high complication rate. The largest glycemic control status group was of individuals with fair control (either consistent or oscillating), who had their HbA1c levels in an intermediate range (between 7.1% and 9%). The overall complication rate in this group of 65 (32.5%) participants was in the intermediate range (Table 4).

**Table 4:** Baseline and follow-up complications in participants (total n=314) CAD: Coronary artery disease

Parameter	Optimally controlled and consistent	Fairly controlled and consistent	Fairly controlled and oscillating	Poorly controlled and oscillating	Poorly controlled and consistent
No. of participants	62	139	61	9	43
Presence of complications at baseline					
Retinopathy	1 (1.61%)	1 (0.71%)	3 (4.91%)	0	2 (4.65%)
Neuropathy	6 (9.67%)	10 (7.19%)	3 (4.91%)	1 (11.11%)	3 (6.97%)
Nephropathy	3 (3.22%)	2(1.43%)	4 (6.55%)	0	2 (4.65%)
Known CAD/Stroke	1 (1.61%)	5 (3.58%)	5 (8.18%)	0	4 (9.29%)
New-onset complications					
Retinopathy	2 (3.22%)	12 (8.63%)	4 (6.55%)	2 (22.22%)	4 (9.30%)
Neuropathy	3 (4.83%)	10 (7.19%)	4 (6.55%)	0	3 (6.97%)
Nephropathy	2 (3.22%)	3 (2.15%)	4 (6.55%)	0	1 (2.32%)
Known CAD/Stroke	3 (4.83%)	6 (4.31%)	4 (6.55%)	3 (33.33%)	2 (4.65%)
Any new complication	10 (16.12%)	34 (24.46%)	13 (21.31%)	5 (55.56%)	10 (23.25%)
Total with a complication	17 (24.41%)	44 (31.65%)	21 (34.42%)	6 (66.67%)	16 (37.20%)

## Discussion

This study highlights that only 20% of diabetic patients achieved optimal glycemic control, while 80% had suboptimal control. Our study shows that HbA1c targets were not met for most diabetic patients in our tertiary care hospital, indicating unsatisfactory glycemic control. Comparable findings of poor glycemic control have been demonstrated in studies conducted in various other developing countries [9,10]. Factors contributing to poor glycemic control include inadequate diabetes education, high-carbohydrate diets, and low physical activity levels [11]. In contrast, better glycemic control in developed countries such as Japan and Germany may stem from higher literacy rates and better disease understanding and awareness [12,13].

In this study, younger individuals, overweight/obese patients, females, and those with both hypertension and diabetes exhibited higher HbA1c values; however, these differences were not statistically significant except in females. Among females, social factors may influence glycemic control, likely due to a lack of empowerment and decision-making. Additionally, patients with a longer duration of diabetes and those using insulin showed poorer glycemic control, but this finding also lacked statistical significance. Interestingly, in our study, older patients have better glycemic control than younger patients. This may be due to extended family arrangements, where the involvement of family members may enhance adherence among elderly patients [14]. However, the association between age and glycemic control was not statistically significant in our study. Similarly, no significant association between age and glycemic control achievement was observed in other studies, too [9,13].

One study conducted in Hong Kong revealed an association between longer diabetes duration and more complex treatment protocols with poorer glycemic control in patients [15]. The longer duration of diabetes has a detrimental impact on glycemic control, likely due to the progressive decline in insulin secretion over time as a consequence of beta-cell dysfunction [16]. Drug utilization patterns also affect outcomes, with monotherapy generally achieving better control than combination therapies. In the current study, a combination of metformin and sulfonylurea was widely used. Patients receiving insulin therapy exhibited higher mean HbA1c levels compared to those on monotherapy or combination oral antidiabetic drug therapy. Individuals with a larger duration or with poorly controlled T2DM are more likely to be on additional oral hypoglycaemic agents. Similarly, insulin is added for individuals with poor control. Significant differences were noted among the three types of drug treatment patterns, consistent with findings from other studies [17]. Notably, adherence to prescribed regimens emerged as a critical determinant of glycemic control, emphasizing the need for improved patient education and adherence strategies. Even a modest increase in adherence, by 10%, has been shown to have a reduction in HbA1c values by 0.16% [18].

Overall, the distribution of variables associated with glycemic control did not significantly differ across categories. Some patients consistently maintained control, others remained uncontrolled, while the majority fell in between. This suggests that treatment effectiveness and glycemic control are influenced not only by age, sex, BMI, duration of diabetes, comorbidities, and medication patterns but also crucially by patient adherence to treatment and the efficacy of prescribed therapies. Furthermore, pharmacogenomics, an area largely unexplored in diabetes and numerous other medical conditions, emerges as a significant determinant affecting glycemic control, highlighting the variability in individual responses to medications. This emphasizes the need for further research into pharmacogenomic factors to optimize diabetes management strategies. Hence, genetics may be the scope of a future study.

Strengths and limitations

This study's strengths include the large sample size and real-world data, study design and longitudinal nature, and inclusion of GV and multi-faceted analysis. As for limitations, first, confounding variables such as dietary habits and precise quantification of sugar intake were not accounted for, primarily due to the challenges associated with obtaining accurate data in these domains. Second, there was variability in the time intervals between the initial and subsequent HbA1c measurements across patients, which may affect the results. Third, some answers to the questionnaire depend on the memory of respondents, which may have resulted in recall bias. Fourth, this study was conducted only among T2DM patients attending a tertiary care government hospital, which may not be representative of the overall type 2 diabetes population. Finally, selection bias and limited generalizability are also limitations.

## Conclusions

In summary, factors such as age, sex, BMI, duration of DM, comorbidities, and pattern of drug utilization are not the only predictors of glycemic control. Other factors, such as consistent adherence, efficacy of the treatment prescribed, also impact good glycemic control. As diabetes progresses as a chronic condition, complications tend to increase, necessitating more complex drug therapies over time. Complications of oscillating/variable glycemic control were the same as those of poorly controlled, despite the oscillator having a lower mean HbA1c value. Those with optimal control and lower long-term GV have better outcomes than all other groups. Control of HbA1c needs to be consistent so that long-term GV is also low.
